# The gut microbiome correlates with conspecific aggression in a small population of rescued dogs *(Canis familiaris)*

**DOI:** 10.7717/peerj.6103

**Published:** 2019-01-09

**Authors:** Nicole S. Kirchoff, Monique A.R. Udell, Thomas J. Sharpton

**Affiliations:** 1 Department of Microbiology, Oregon State University, Corvallis, OR, United States of America; 2 Department of Animal and Rangeland Science, Oregon State University, Corvallis, OR, United States of America; 3 Department of Statistics, Oregon State University, Corvallis, OR, United States of America

**Keywords:** Gut microbiome, Aggression, Fecal microbiota, Dog, Gut-brain axis

## Abstract

Aggression is a serious behavioral disorder in domestic dogs that endangers both dogs and humans. The underlying causes of canine aggression are poorly resolved and require illumination to ensure effective therapy. Recent research links the compositional diversity of the gut microbiome to behavioral and psychological regulation in other mammals, such as mice and humans. Given these observations, we hypothesized that the composition of the canine gut microbiome could associate with aggression. We analyzed fecal microbiome samples collected from a small population of pit bull type dogs seized from a dogfighting organization. This population included 21 dogs that displayed conspecific aggressive behaviors and 10 that did not. Beta-diversity analyses support an association between gut microbiome structure and dog aggression. Additionally, we used a phylogenetic approach to resolve specific clades of gut bacteria that stratify aggressive and non-aggressive dogs, including clades within *Lactobacillus*, *Dorea*, *Blautia*, *Turicibacter,* and *Bacteroides*. Several of these taxa have been implicated in modulating mammalian behavior as well as gastrointestinal disease states. Although sample size limits this study, our findings indicate that gut microorganisms are linked to dog aggression and point to an aggression-associated physiological state that interacts with the gut microbiome. These results also indicate that the gut microbiome may be useful for diagnosing aggressive behaviors prior to their manifestation and potentially discerning cryptic etiologies of aggression.

## Introduction

Domestic dogs (*Canis familiaris*) have coexisted with humans for over 14 thousand years (Nobis, 1979), and remain among the most popular companion animals, especially in the Western world where millions can be found living in human homes (American Pet Products Association, 2014). Even larger populations of free-roaming and village dogs can be found living among humans around the world (Coppinger & Coppinger, 2001). In recent years, dogs have been studied for their capacity to form strong bonds with humans and other species, resulting in a range of prosocial, cooperative, and communicative behaviors (Udell & Wynne, 2008). However, dog aggression towards humans, other dogs, or other animals remains a common behavioral problem (Bamberger & Houpt, 2006) that can pose serious risks to animals, owners, and other humans including neighbors, friends, or veterinary staff. Aggressive interactions, especially those involving bites, may lead to physical harm, psychological trauma, disease transmission, or even fatality in bitten humans and other dogs (Overall & Love, 2001; Hampson et al., 2009; Brooks, Moxon & England, 2010; Ji et al., 2010). Aggressive behavior also poses risks to the aggressor dog, as aggression is a common reason for relinquishment to animal shelters, where poor progress on mitigation of the behavior, assuming the shelter is even equipped to intervene, often leads to euthanasia (Salman et al., 2000). Consequently, understanding the factors and mechanisms responsible for dog behaviors that are incompatible with success in anthropogenic environments has much potential to benefit both species.

Dog aggression is often divided into categories, including dominance aggression, fear aggression, food or possessive aggression, and territorial aggression (Blackshaw, 1991; Houpt, 2006; Lockwood, 2016) based on the form of the behavior and the identified or presumed context or consequences associated with specific aggressive acts. However, the factors that predict aggression in one dog, but not in another, under similar conditions (for example, in a standard behavior evaluation) are less well understood. Current research suggests that environmental, experiential, and owner specific variables are important predictors of aggression in dogs (Roll & Unshelm, 1997; Hsu & Sun, 2010). However, underlying biological correlates including genetics, sex, hormone levels, neuter status, nutrition, and neurological health have also been identified (Sherman et al., 1996; DeNapoli et al., 2000; Duffy, Hsu & Serpell, 2008; Rosado et al., 2010). While behavior modification or environmental change can significantly reduce aggressive behavior in at least some contexts (Sherman et al., 1996; Mohan-Gibbons, Weiss & Slater, 2012), underlying physiological mechanisms including pain, elevated stress levels, reduced thresholds for aggression, or impulsivity could impede behavioral treatment or lead to resumption of the behavior if left unidentified. Therefore, further elucidating the physiological underpinnings of aggression in dogs may be critical to mitigating aggressive behavior, especially for situations where other treatment and training options are ineffective on their own. The limited research in this area shows that aggression associates with high levels of cortisol and low levels of serotonin (5HT) (Rosado et al., 2010; León et al., 2012; Roth et al., 2016). Stress in dogs is often detected by measuring cortisol levels and is thought to be a component associated with behavioral problems such as anxiety as well as aggression (Rooney, Clark & Casey, 2016). Accordingly, many dogs diagnosed with aggression are also diagnosed with anxiety (Bamberger & Houpt, 2006). Behaviors associated with anxieties in dogs include increased heart rate, trembling, increased salivation, pacing, circling, transient anorexia, inappropriate elimination, excessive vocalization, destructiveness, and restlessness (Stelow, 2018). Dogs with anxiety may also present with aggressive behaviors such as lunging (Stelow, 2018). There remains much to learn about the underlying causes of aggressive behavior, which limits the development of new preventative strategies, diagnostics, and therapeutic interventions.

Emerging evidence suggests that the gut microbiome may interact with mammalian physiology to influence behavior (Cryan & O’Mahony, 2011; Mayer et al., 2014; Foster et al., 2016). These interactions include aspects of physiology that are relevant to mammalian aggression. For example, treatments with a broad-spectrum antibiotic disrupted the gut microbiome and decreased aggressive behavior in Siberian hamsters (Sylvia et al., 2017). Additionally, germ-free and specific-pathogen free mice exhibit different anxiety levels (Heijtz et al., 2011; Neufeld et al., 2011). Other studies have found that specific strains of bacteria (i.e., probiotics) improve the health of the host by modulating anxiety phenotypes and stress hormones such as glucocorticoids. For example, administration of *Lactobacillus rhamnosus* (JB-1) reduced anxiety related behavior in mice, and *Bacteroides fragilis* NCTC 9343 improves anxiety-like behavior in a mouse model known to exhibit anxiety-like behaviors (Bravo et al., 2011; Hsiao et al., 2013). Moreover, gut bacteria can produce neuroactive substances, such as precursors of monoamine neurotransmitters that act on the gut-brain axis to potentially impact behavior, including anxiety (Heijtz et al., 2011; Evrensel & Ceylan, 2015; Carabotti et al., 2015; O’Mahony et al., 2015). For example, the gut microbiome produces tryptophan, which impacts host serotonin levels and behaviors linked to serotonergic neurotransmission (O’Mahony et al., 2015; Yano et al., 2015). Several studies show an inverse relationship between the serotonin metabolite, 5-hydroxyindoleacetic acid (5-HIAA), and aggressive behaviors (Coccaro et al., 2015). Collectively, these observations indicate that the gut microbiome and aggressive behavior may be linked in mammals.

To date, no studies have investigated the association between the gut microbiome and aggression in dogs, which is a first necessary step towards ultimately ascertaining whether the gut microbiome mediates aggression. Prior work points to a potential interaction between the microbiome and canine aggression. For example, diet is a strong modulator of gut microbial composition in many animals (David et al., 2013) and specific dietary components are associated with aggression including diets that reduce aggressive behaviors in dogs (DeNapoli et al., 2000; Re, Zanoletti & Emanuele, 2008). Additionally, the canine gut microbiome associates with other health conditions such as inflammatory bowel disease and acute diarrhea (Suchodolski et al., 2012) leading to discomfort or pain that could also contribute to irritability or aggression. Here, we conducted an exploratory analysis of fecal samples originating from a small shelter-housed population of pit bull type dogs seized from organized dogfighting to determine if canine aggression could be predicted based on the composition of the gut microbiome.

## Materials and Methods

### Sample collection

A single fecal sample was collected from the kennel of each of 31 pit bull type dogs residing at a temporary shelter while in protective custody. The inner core of the feces was sampled in order to minimize potential bacterial contamination given that the feces were in contact with the kennel floor. This population served as the focus of this pilot study because it enabled control over as many factors as possible, including breed type, environment, diet, and medical care, while providing access to a population with a relatively more frequent aggressive phenotype compared to typical populations. Upon intake into the shelter and prior to the initiation of this study, an animal welfare agency catalogued various parameters of each individual, which were used in this study’s analysis as covariate data (Table S1). Animal welfare employees collected feces using aseptic technique within an hour of defecation and immediately froze them at −18 °C to −20 °C to fix bacterial growth and preserve the DNA content. Fecal samples were shipped to Oregon State University and stored at −20 °C. Thirty of the dogs were on a diet of Iams Proactive Health minichunks adult kibble (chicken-based formula) and one dog was on a diet of Iams Puppy. Fourteen males and 17 females received a behavior evaluation conducted by the animal welfare agency shortly after intake that categorized these dogs as intraspecifically aggressive (*n* = 21) or non-aggressive (*n* = 10). Aggressive dogs displayed aggression during one of three scenarios: an introduction to a life-size dog plush, introduction to a dog of the same sex behind a barrier, and introduction to a dog of the same sex without a barrier. Aggressive displays toward the life-size dog plush included growling, snarling, biting, biting and holding, biting and shaking combined with tense behavior inconsistent with object play, and aggressive displays toward the same sex dogs included growling and lunging, lunging and snarling, climbing on withers and growling, attempting to bite, and biting. Non-aggressive dogs did not display aggression towards the dog plush or another dog (Text S1). Data from these evaluations were sent to Oregon State University along with the stool samples for analysis. With the exception of the collection and processing of fecal material, this study did not involve any manipulation of, measurement of, or contact with dogs that had not already occurred.

### Ethical statement

No animal subjects, animal handling, or study specific animal interactions were required for the purpose of this study. Dog fecal samples were collected from shelter kennels after natural deposit. Previously collected behavioral data from the animal welfare agency’s records were used in analysis. Therefore, this study was determined to be exempt from institutional animal care and use review by Oregon State University’s ethical review board.

### Fecal DNA extraction and 16S sequencing

DNA was extracted from fecal samples using the QIAGEN DNeasy^®^ PowerSoil^®^ DNA isolation kit (QIAGEN, Germantown, MD USA) as per manufacturer instruction with the exception of an additional heat incubation of 10 min at 65 °C immediately before the bead beating step. The 16S rRNA gene was amplified from the extracted DNA with PCR and primers designed to target the V4 region (Caporaso et al., 2012). Amplicons were subsequently quantified using the Qubit^®^ HS kit (Thermo Fisher, Waltham, MA USA) and then pooled and cleaned using the UltraClean^®^ PCR clean-up kit (MO BIO, Carlsbad, CA USA). These cleaned amplicons were then sequenced on an Illumina MiSeq (v3 chemistry) instrument. This sequencing generated 3.31 million 150 bp single end reads (median reads per sample = 78,272).

### Bioinformatic and statistical analyses

The QIIME (v1.8.0) bioinformatics pipeline was used to quality control raw sequences as well as quantify the diversity of microorganisms isolated from the fecal samples. The Illumina-generated sequences were demultiplexed and quality filtered (i.e., sequences with a Phred quality score less than 20 were removed) with the QIIME script split_libraries_fastq.py. The pick_open_reference_otus.py script then assigned sequences to Operational Taxonomic Units (OTUs) based on the alignment of sequences to the Greengenes (v13_8) reference database using a 97% similarity threshold with the UCLUST algorithm (v1.2.22). With the core_diversity_analysis.py script, samples were subject to rarefaction through random sub-sampling of sequences at a depth of 40,000 reads, which corresponded to the lowest sequencing depth obtained across samples. The BIOM table generated from the core_diversity_analysis.py script was imported into R and potentially spurious OTUs were filtered by removing those that (1) were found in fewer than three samples and (2) were observed fewer than 20 times across all samples from all subsequent analyses. The resulting OTU matrix was subsequently processed using the beta_diversity.py script to calculate the weighted and unweighted UniFrac distances between all pairs of samples (Lozupone & Knight, 2005). Alpha diversity was calculated in R (v3.2.3) using the diversity function in the vegan package (v2.3-3).

Intersample similarity was visualized using principal coordinates analysis (PCoA) based on the Bray–Curtis dissimilarity index using the vegan (v2.3-3) package in R (v3.2.3). The association between sample covariates, including dog aggression, and intersample similarity was quantified with the envfit function in the vegan package. Kruskal–Wallis tests, as implemented by the coin package (version 1.1-2), were used to identify OTUs and phylotypes that stratify samples by covariate factors. Phylogenetic clades that associate with aggression were identified by assembling a reference-guided 16S sequence phylogeny via FastTree as previously described (O’Dwyer, Kembel & Sharpton, 2015), using Claatu to resolve monophyletic clades that are conserved in aggressive or non-aggressive dogs (FDR <  0.05) (Gaulke et al., 2018), and Kruskal–Wallis tests to ascertain if these conserved clades are differentially abundant across these populations. The taxonomy of these clades was determined by identifying the most resolved taxonomy label that is shared among all members of the clade. Multiple tests were corrected using the qvalue package (version 2.2.2). Phylotypes or clades with a *p*-value less than 0.05 and a *q*-value less than 0.2 were designated as those that stratify samples.

## Results

**Figure 1:**
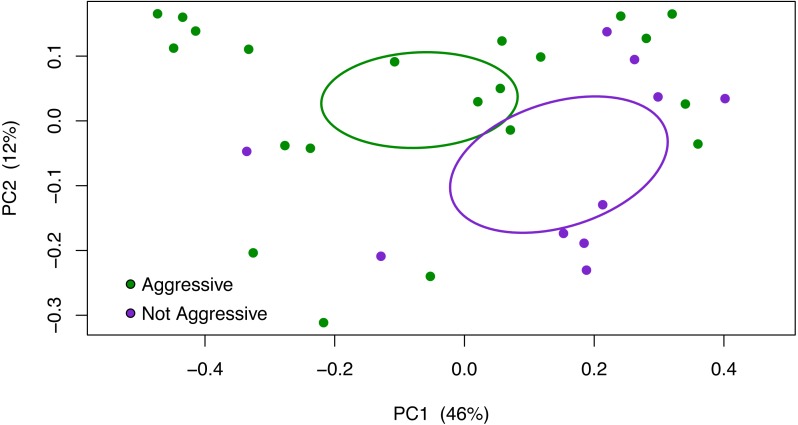
Aggressive and non-aggressive dogs differ in beta-diversity using the weighted UniFrac metric. Visualization of the phylogenetic differences in fecal microbiota of aggressive (green) and non-aggressive (purple) dogs using principal coordinates analysis (PCoA) of OTU abundances and weighted UniFrac distance. The separation between aggressive and non-aggressive samples in the PCoA plot was confirmed with an environmental fit analysis (*p* = 0.0250, *R*^2^ = 0.1297), which supports aggression status as being a variable that is separating the microbial composition of the samples. The gut microbiome structure of aggressive and non-aggressive dogs is also significantly different with the weighted UniFrac metric using PERMANOVA (*p* = 0.0346, *R*^2^ = 0.0349). Ellipses are based on 95% confidence intervals and standard error.

To determine possible differences in gut microbial composition between aggressive and non-aggressive dogs, we compared stool microbiomes that were sampled from 21 aggressive dogs and 10 non-aggressive dogs. A Principal Coordinates Analysis (PCoA) using the weighted UniFrac metric shows separation of the aggressive and non-aggressive samples based on 95% confidence interval ellipses (Fig. 1; Figs. S1; S2). The separation between aggressive and non-aggressive samples in the PCoA plot was confirmed by environmental fit (*p* = 0.0250, *R*^2^ = 0.1297) and PERMANOVA (*p* = 0.0346, *R*^2^ = 0.0349) analyses. Alternative measures of beta-diversity marginally support these results. For example, using a Bray–Curtis dissimilarity metric finds a similar trend (PERMANOVA, *p* = 0.0957, *R*^2^ = 0.0573). Other study covariates were tested for their association with the fecal microbial composition. Dog age did not associate with microbial composition (weighted UniFrac, PERMANOVA, *p* = 0.1763, *R*^2^ = 0.0652). Conversely, the sex of the dogs did associate with microbial composition when using unweighted UniFrac (PERMANOVA, *p* = 0.0400, *R*^2^ = 0.0652), but not when using weighted UniFrac (PERMANOVA, *p* = 0.1424, *R*^2^ = 0.0582). Unlike the differences in beta-diversity between aggressive and non-aggressive dogs, no significant differences were detected in alpha diversity based on the Shannon index when comparing behavioral groups (*p* = 0.5258).

**Figure 2:**
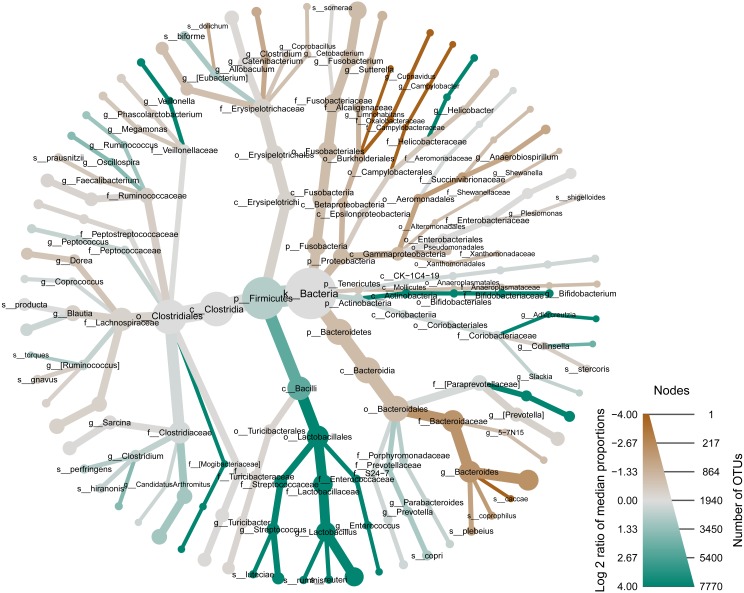
Many of the most relatively abundant phylotypes in our dog fecal samples are significantly different across aggressive and non-aggressive dogs. A metacoder (Foster, Sharpton & Grünwald, 2017) heattree illustrates the variation in microbiome phylotypes between the aggressive and non-aggressive dog populations. Nodes in the heattree correspond to phylotypes, as indicated by node labels, while edges link phylotypes in accordance to the taxonomic hierarchy. Node sizes correspond to the number of OTUs observed within a given phylotype. Colors represent the log fold difference of a given phylotype’s median relative abundance in the aggressive dogs as compared to the non-aggressive dogs. Specifically, darker green represents higher relative abundance of aggressive OTUs and darker brown represents higher relative abundance of non-aggressive OTUs.

**Figure 3:**
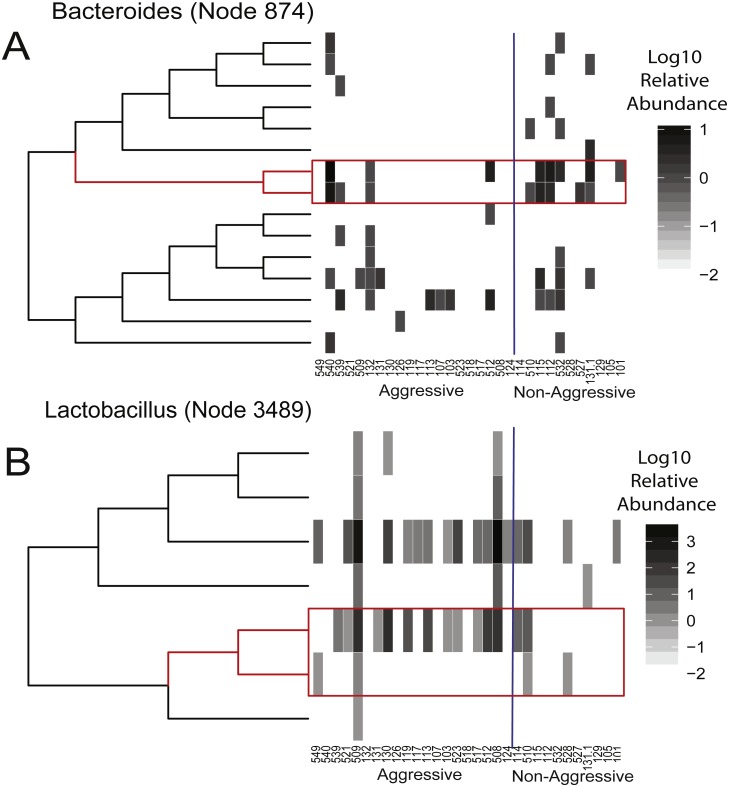
The abundance of monophyletic clades within phylotypes stratify aggressive and non-aggressive dogs. (A) illustrates a subtree within the *Bacteroides* phylotype containing node 874 (red branches), which is a monophyletic clade that is both common to and relatively more abundant amongst the non-aggressive individuals than the aggressive individuals. The heat map adjacent to this subtree illustrates the log10 relative abundance of each lineage in this subtree across the individuals subject to our investigation. The red rectangle highlights the relative abundance of the lineages within node 874. The vertical blue line separates aggressive and non-aggressive individuals. (B) illustrates a similar subtree, but in this case, it has been extracted from within the *Lactobacillus* phylotypes and highlights a monophyletic clade (node 3489) that is common to and relatively more abundant amongst the aggressive dogs.

The bacterial phylotypes that were observed across the dog fecal samples were compared between behavioral groups to resolve those phylotypes that vary in association with aggression (Fig. 2). Firmicutes, Fusobacteria, Bacteroidetes, and Proteobacteria were the dominant phyla in all fecal samples. The relative abundances of these predominant phyla also significantly differed across aggressive and non-aggressive dogs (*p* < 0.05, *q* <  0.1). Specifically, Proteobacteria and Fusobacteria manifested higher relative abundance in non-aggressive dogs, while Firmicutes was relatively more abundant in aggressive dogs. These trends were driven by variation in a small number of more granular phylotypes (Fig. 3). The family Lactobacillaceae was more abundant in aggressive dogs, while the family Fusobacteriaceae was more abundant in non-aggressive dogs (*p* < 0.05, *q* < 0.2). Consistently, the genus *Lactobacillus* was more abundant in aggressive dogs, while the genus *Fusobacteria* was more abundant in non-aggressive dogs (*p* < 0.05, *q* < 0.2). Additional separation between aggressive and non-aggressive dogs was observed at the OTU level. Specifically, seven OTUs significantly differed between aggressive and non-aggressive dogs (*p* < 0.05, *q* < 0.1), including four OTUs from the genus *Dorea*, two OTUs from the genus *Lactobacillus*, and one OTU from *Turicibacter*. All of the phylotypes and OTUs that significantly associated with aggression are included in Table S2 (phylotypes) and Table S3 (OTUs).

To better resolve taxa that stratify aggressive and nonaggressive dogs, we used a phylogenetic approach that defines taxa as monophyletic clades of bacteria that are prevalently observed across members of the aggressive or nonaggressive populations. These clades represent evolutionary groupings of bacteria that often correspond to intermediate levels of taxonomy (e.g., between species and genus) that are defined by the shared ancestry and ecology of clade members. Moreover, by focusing on prevalent clades, which are those that are observed in more individuals within a population than expected by chance (Gaulke et al., 2018), we are able to resolve bacterial taxa that are especially common to at least one population. This property of a high prevalence of behavior-stratifying gut microbes may be a desirable characteristic when searching for potentially diagnostic indicators of aggression status.

Of the 578 clades that are prevalent in either aggressive or non-aggressive dogs, 96 significantly differ in abundance between the two populations (*q* < 0.2). Of these clades, 39 have a mean relative abundance that is significantly higher in the gut microbiomes of aggressive dogs, while 57 have a higher relative abundance in non-aggressive dog microbiomes. A complete list of clades that associate with behavior can be found in Table S4. Of particular note is our finding that nine clades with the genus *Bacteroides* are elevated in the gut microbiomes of non-aggressive dogs compared to aggressive dogs. This finding indicates that the relative abundance of these lineages within *Bacteroides* may predict aggression status and that their depletion may contribute to aggression. We also find that the genus *Lactobacillus* contains 25 clades that are relatively abundant in aggressive canines. Similar patterns are observed for clades within the family Paraprevotellaceae. These observations indicate that aggression may be associated with an increase in specific lineages within *Lactobacillus* and Paraprevotellaceae and they may express traits that interact with aggression-associated aspects of canine physiology. Moreover, we find that the genus *Turicibacter* contains both aggression-elevated and aggression-depleted clades (Fig. S3), indicating that descendants of this genus may have recently evolved traits that contribute to their differential association with canine behavior.

## Discussion

Accumulating evidence indicates that the gut microbiome acts as an agent of the nervous system and influences affective disorders such as anxiety and depression (Clapp et al., 2017). However, it is unknown if the gut microbiome similarly relates to animal aggression. Our exploratory analysis of a population of rescued, sheltered-housed dogs links the composition of the gut microbiome to conspecific aggression in canines. While this associative study cannot disentangle cause and effect, it holds important implications for clinical practices surrounding canines, as its results indicate that: (a) the gut microbiome may contribute to aggression or its severity, and that manipulation of the microbiome (e.g., by probiotic administration) may alleviate the behavior; (b) the physiology of aggressive dogs results in different gut microbiome compositions, which indicates that the microbiome may facilitate predictive diagnosis of aggressive behavior and preventative intervention; or (c) aggression and the gut microbiome are similarly associated with a cryptic physiological or environmental covariate, such as inflammation or cortisol levels, which may help discern the physiological underpinnings of canine aggression. Future studies should build upon this exploratory investigation to discern the mechanisms underlying the relationship between canine aggression and the gut microbiome.

Our investigation finds that the composition of the gut microbiome differs between aggressive and non-aggressive dogs in the population that we studied. The rescued, shelter-housed dogs included in this investigation proved useful for this study because they included aggressive and non-aggressive individuals and were taken into the shelter at the same time, maintained in the same facility, mostly exposed to the same diet, and generally of consistent breed type. Despite our attempt to homogenize the sources of variation amongst these dogs, we observed extensive variation in the composition of the gut microbiome within each behavioral cohort. This intra-cohort variation indicates that the stool samples we studied are subject to cryptic factors that associate with microbiome composition (e.g., early life history (Rodríguez et al., 2015)). This is unsurprising given that individuals living outside of a laboratory setting (including pet and shelter dogs, as well as humans) are subject to genetic and environmental diversity that cannot fully be controlled for. That said, the identification of significant differences between these populations under naturalistic conditions heightens the applied value of these findings. We were able to measure other factors that may influence the gut microbial composition of these dogs besides aggression and found that dog sex partially explains the inter-individual variation in the unweighted UniFrac dissimilarity of gut microbiome samples. Considering this result alongside the finding that aggression status links to the weighted UniFrac dissimilarity of the same samples indicates that, of the covariates measured in this study, sex explains the types of microbes that are present in the gut of these dogs while aggression status explains which of the microbes dominates their gut community. These observations suggest that dog sex, aggression, and gut microbiome composition are intertwined, and align with prior work that observed sex-dependent effects on how disruption of the gut microbiome affects animal aggression (Sylvia et al., 2017). Also, because prior experiences can impact the gut microbiome and because we do not know the prior experiences of these individuals, future studies may find that aggression is not linked to the microbiome in other dogs. Such a finding would indicate that there are contextual dependencies underlying the aggression-microbiome connection observed in this study. Additionally, researchers should observe if there is a consistent connection between canine aggression and microbial composition while correcting for possible confounding variables such as age and diet, measuring additional forms of aggression, such as aggression towards humans, and studying greater numbers of pit bull breed type dogs or including different dog breeds in study populations. Future efforts should consider larger populations and measure more diverse covariates per individual to disentangle the properties that influence the gut microbiome’s apparent relationship with aggression.

Several taxa found in our study were also observed in other canine gut microbiome studies. For example, the most abundant phyla, Firmicutes, Fusobacteria, Bacteroidetes, and Proteobacteria, were also dominant in fecal samples from previous canine gut microbiome studies (Deng & Swanson, 2015). Additionally, several taxa significantly differ in their relative abundance between aggressive and nonaggressive dogs. For instance, we find that that lineages within the genus *Bacteroides* are elevated in non-aggressive dogs, which might be expected given that species within this genus, such as *Bacteroides fragilis*, have been shown to modulate mammalian behavior in prior investigations (Hsiao et al., 2013). Moreover, the genus *Dorea* elevates in non-aggressive dogs compared to aggressive dogs, which is notable because *Dorea* manifests a reduced abundance in dogs afflicted with inflammatory bowel disease (Jergens et al., 2010) and other enteropathies (Suchodolski, 2011), and because psychological disorders are frequently comorbid with gastrointestinal inflammation (Bannaga & Selinger, 2015; Clapp et al., 2017). However, our observations of which taxa stratify these cohorts are not always consistent with prior investigations of microbial taxa that associate with mammalian behavior. As an example, we find that members of *Lactobacillus* are more abundant in the gut microbiomes of aggressive dogs, which might defy expectations given that prior research of specific strains of *Lactobacillus rhamnosus* have been found to reduce stress-associated corticosterone levels and anxiety related behavior in mice and is known to produce GABA neurotransmitters (Bravo et al., 2011). Similarly, the genus *Fusobacterium* is typically thought to elicit pro-inflammatory effects inside the gut (Bashir et al., 2016); here, we find that *Fusobacterium* is more abundant in the stool of non-aggressive dogs. That said, it is challenging to determine the physiological role of specific microbiota from 16S sequences given that an organism’s interaction with its host may be context dependent (Schubert, Sinani & Schloss, 2015) and may rapidly diversify (Conley et al., 2016). Indeed, our analysis of monophyletic clades of gut bacteria that associate with aggression finds that closely related clades can manifest opposite patterns of association with behavior, such as that of *Turicibacter*. Additionally, the limited population size may challenge the discovery of taxa that statistically stratify cohorts. Despite this, these taxa represent compositional distinctions between aggressive and non-aggressive dogs in our population. Further study of their physiological role may help clarify whether or how they influence canine aggression as well as their probiotic suitability and therapeutic capacity towards alleviating aggression in dogs.

## Conclusions

Our results indicate that there are statistical associations between aggression status and the gut microbiome. For example, microbial composition differs based on aggressive and non-aggressive evaluations. Additionally, the relative abundances of specific bacterial taxa and lineages are different across aggressive and non-aggressive groups. These observations are important because they indicate that either (a) aggressive dogs manifest physiological conditions in the gut that influence the composition of the gut microbiome, (b) the composition of the gut microbiome may influence aggressive behavior, or (c) that aggressive dogs are subject to some biased covariate relative to non-aggressive dogs that also influences the gut microbiome. Future studies should seek to confirm that these findings are consistent in additional populations of dogs, and seek to discriminate between these possibilities. Additionally, future studies should expand the size of the populations being studied, measure a diverse array of physiological covariates to tease out aggression-specific effects and discern mechanisms of interactions, identify and test specific bacterial strains as probiotics that could alleviate aggression, and consider using metagenomic analyses to deduce the potential functional role of the microbiome in these interactions.

Ultimately, our results indicate that the composition of the gut microbiome associates with conspecific canine aggression in this group of dogs. These results pave the way for future investigations to ascertain whether similar results are seen in other dog populations and if the microbiome can be used to develop diagnostics, preventative strategies, and therapeutics of aggression.

##  Supplemental Information

10.7717/peerj.6103/supp-1 Table S1 Co-variate data for all dogs included in our study

10.7717/peerj.6103/supp-2 Table S2 Statistics and additional information about phylotypes of interest identified by Kruskal–Wallis tests Includes *p*-values from Kruskall-Wallis tests, *q*-values from multiple test corrections, taxon ranks, bacterial taxa, and the magnitude of the difference those taxon abundances of taxa observed in aggressive over non-aggressive dogs.

10.7717/peerj.6103/supp-3 Table S3 Statistics and additional information about OTUs of interest identified by Kruskal-Wallis tests Includes *p*-values from Kruskal-Wallis tests, *q*-values from multiple test corrections, OTU taxonomy strings, OTU identification numbers, and the magnitude of the difference in OTU abundances of OTUs observed in aggressive over non-aggressive dogs.

10.7717/peerj.6103/supp-4 Table S4 Statistics and additional information about clades of interest identified by Kruskal-Wallis tests Includes clade identification numbers, *p*-values from Kruskal-Wallis tests, *q*-values from false discovery rate adjusted p-values, bacterial taxonomy strings for each clade, and the magnitude of the difference in clade abundances of clades observed in aggressive over non-aggressive dogs.

10.7717/peerj.6103/supp-5 Figure S1 Weighted UniFrac-based principal coordinates analysis ordination of aggressive and non-aggressive canine fecal microbiomes, axes 1 and 3 Visualization of the phylogenetic differences in fecal microbiota of aggressive (green) and non-aggressive (purple) dogs using principal coordinates analysis (PCoA) of OTU abundances and weighted UniFrac distance across principal coordinates one and three. The gut microbiome structure of aggressive and non-aggressive dogs is significantly different with the weighted UniFrac metric using PERMANOVA (*p* = 0.0346, *R*^2^ = 0.0349), which incorporates all principal coordinates. Ellipses are based on 95% confidence intervals and standard error.

10.7717/peerj.6103/supp-6 Figure S2 Weighted UniFrac-based principal coordinates analysis ordination of aggressive and non-aggressive canine fecal microbiomes, axes 2 and 3 Visualization of the phylogenetic differences in fecal microbiota of aggressive (green) and non-aggressive (purple) dogs using principal coordinates analysis (PCoA) of OTU abundances and weighted UniFrac distance across principal coordinates one and three. The gut microbiome structure of aggressive and non-aggressive dogs is significantly different with the weighted UniFrac metric using PERMANOVA (*p* = 0.0346, *R*^2^ = 0.0349), which incorporates all principal coordinates. Ellipses are based on 95% confidence intervals and standard error.

10.7717/peerj.6103/supp-7 Figure S3 Two clades within Turicibacter that manifest opposite associations with aggression This image is similar to figure 3, except that two clades within a subtree of *Turicibacter* highlighted: node1504, which is common to and more abundant in the aggressive dogs, and node 1573, which is common to and more abundant in the non-aggressive dogs.

10.7717/peerj.6103/supp-8 Supplemental Information 1 A detailed list describing how the animal welfare organization assessed dog aggression

## Additional Information and Declarations

### DNA Deposition

The following information was supplied regarding the deposition of DNA sequences: Oregon State University CGRB, Sharpton Lab Public Repository:
http://files.cgrb.oregonstate.edu/Sharpton_Lab/Papers/Kirchoff_PeerJ_2018/data/raw_16S/.

### Data Availability

The following information was supplied regarding data availability: Sharpton Lab Repository:
http://files.cgrb.oregonstate.edu/Sharpton_Lab/Papers/Kirchoff_PeerJ_2018/.
