# Supplementary figures and images for: The gut microbiome correlates with conspecific aggression in a small population of rescued dogs (Canis familiaris)
Source: PeerJ. 2019 Jan 9;7:e6103. doi: 10.7717/peerj.6103 (PMC6330041; doi:10.7717/peerj.6103)

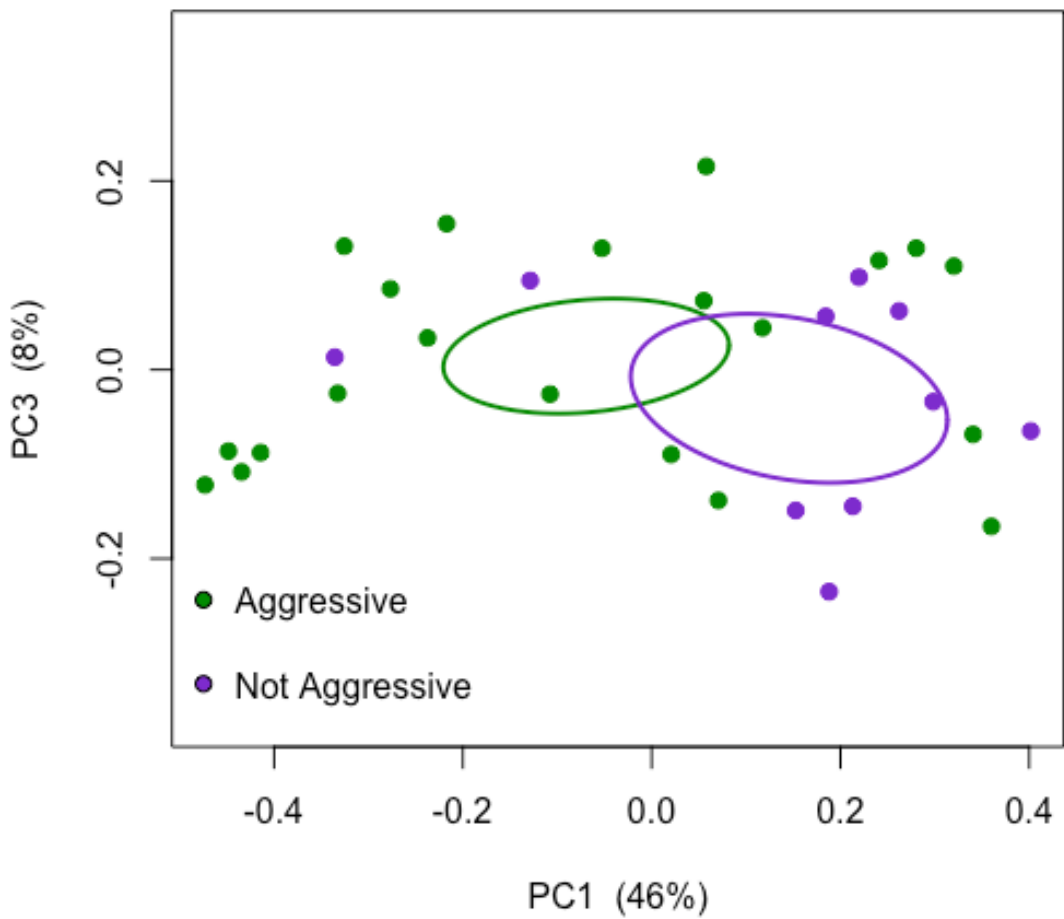

Supplement: Figure S1 — Visualization of the phylogenetic differences in fecal microbiota of aggressive (green) and non-aggressive (purple) dogs using principal coordinates analysis (PCoA) of OTU abundances and weighted UniFrac distance across principal coordinates one and three. The gut microbiome structure of aggressive and non-aggressive dogs is significantly different with the weighted UniFrac metric using PERMANOVA (p = 0.0346, R2 = 0.0349), which incorporates all principal coordinates. Ellipses are based on 95% confidence intervals and standard error. [file peerj-07-6103-s005.pdf]

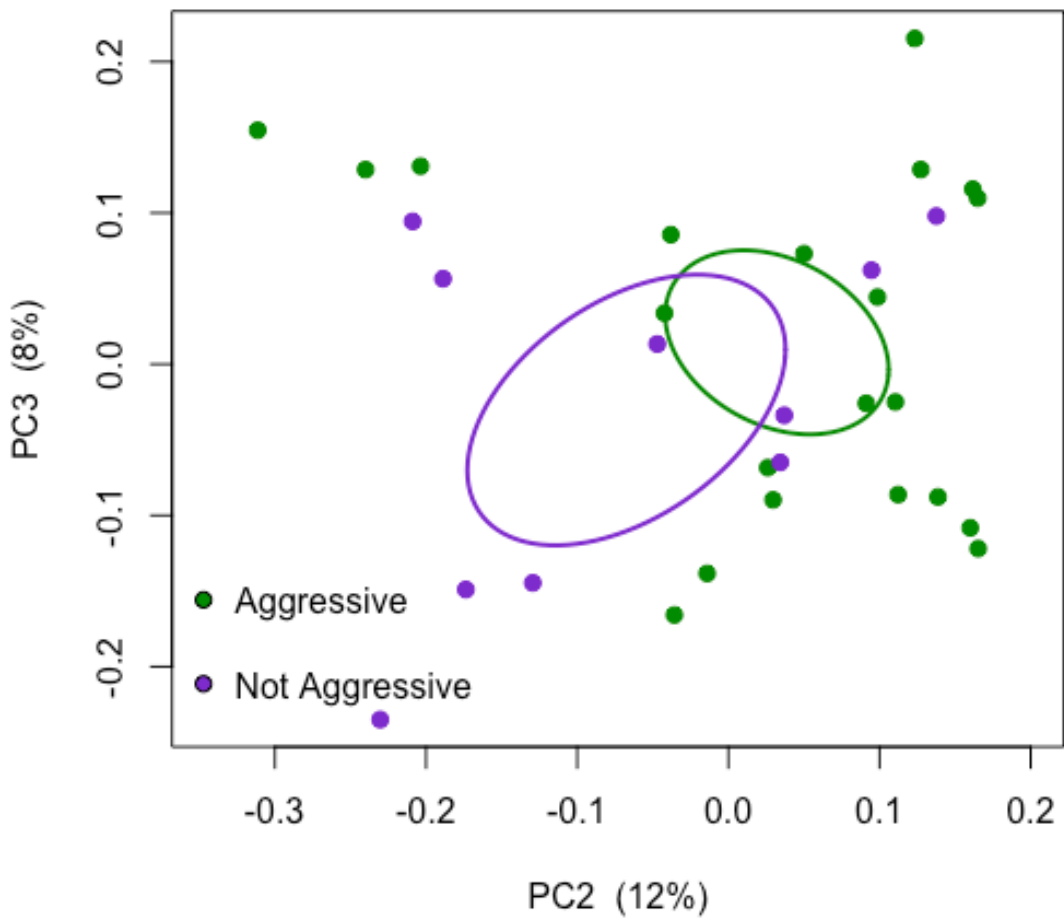

Supplement: Figure S2 — Visualization of the phylogenetic differences in fecal microbiota of aggressive (green) and non-aggressive (purple) dogs using principal coordinates analysis (PCoA) of OTU abundances and weighted UniFrac distance across principal coordinates one and three. The gut microbiome structure of aggressive and non-aggressive dogs is significantly different with the weighted UniFrac metric using PERMANOVA (p = 0.0346, R2 = 0.0349), which incorporates all principal coordinates. Ellipses are based on 95% confidence intervals and standard error. [file peerj-07-6103-s006.pdf]

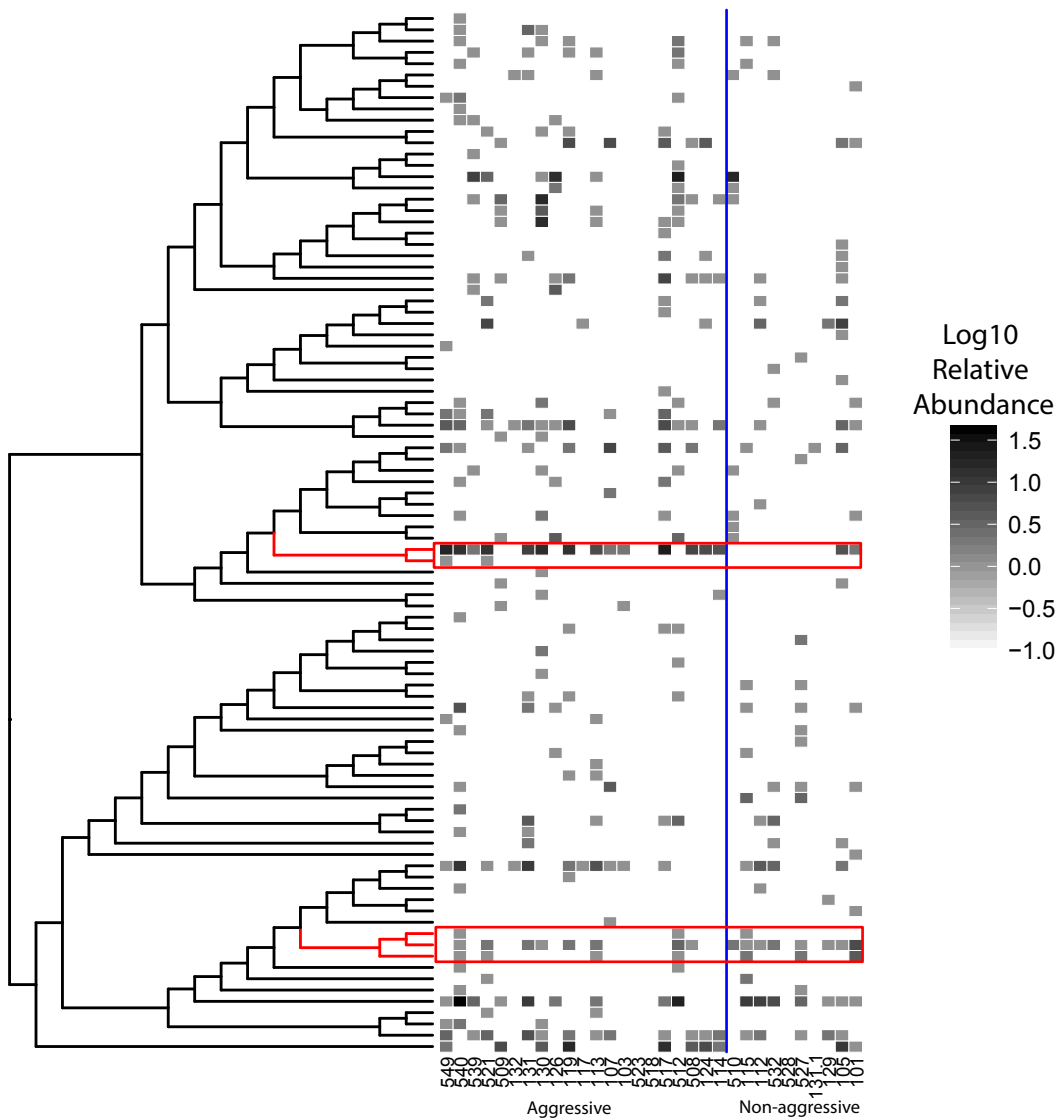

Supplement: Figure S3 — This image is similar to figure 3, except that two clades within a subtree of Turicibacter highlighted: node1504, which is common to and more abundant in the aggressive dogs, and node 1573, which is common to and more abundant in the non-aggressive dogs. [file peerj-07-6103-s007.pdf]
