# Supplementary material for: The gut microbiome correlates with conspecific aggression in a small population of rescued dogs (Canis familiaris)
Source: PeerJ. 2019 Jan 9;7:e6103. doi: 10.7717/peerj.6103 (PMC6330041; doi:10.7717/peerj.6103)
Supplement: Supplemental Information 1 [file peerj-07-6103-s008.docx]

**Supplemental Methods: Canine aggression assessment**

The animal welfare organization used a multistep procedure to assess aggression. Specifically, the following procedure was implemented in the order itemized below.

1. The dog is on a wide collar and leash while attached to an experienced handler. The wide collar is used to prevent damage to the neck if the dog pulls or lunges.
2. The dog is presented with a life-size plush dog that resembles a yellow Labrador retriever.
3. The dog is permitted to interact freely with the plush dog.
4. If the dog exhibits clear-cut displays of aggression toward the plush dog (growling, snarling, biting, biting and holding, biting and shaking, combined with tense body inconsistent with object play), the dog is deemed aggressive and testing is terminated.
5. If the dog does not exhibit clear-cut displays of aggression, the next step is to allow the dog to meet another dog (i.e., a social “stimulus dog” of the same sex) behind a barrier. The barrier is a wire magnum panel so the dogs can see, hear, smell, and even potentially touch. Both dogs are leashed and attached to experienced handlers. If the dog being tested exhibits aggression, testing is discontinued.
6. If no aggression is exhibited, the barrier is removed, the stimulus dog is brought into the room, and the dogs are permitted to greet. If the test dog becomes aggressive before or as the dogs get close to each other, testing is terminated, the dogs are separated, and the test dog is labeled as aggressive.

The outcome of this assessment (i.e., whether aggressive behavior was observed or not) was assigned to each dog by the welfare organization and the final designation was sent to the study team along with associated stool samples for analysis and comparison.
